# An Online Single-Session Cognitive Behavioral Therapy for Depression and Anxiety Associated with Multiple Sclerosis—Pilot Study

**DOI:** 10.3390/bs14070620

**Published:** 2024-07-21

**Authors:** Alina Schenk, Cosmin Octavian Popa, Cristiana Manuela Cojocaru, Ștefan Marian, Smaranda Maier, Ovidiu Lucian Băjenaru, Rodica Bălașa

**Affiliations:** 1 The Doctoral School, George Emil Palade University of Medicine, Pharmacy, Science and Technology, 540142 Targu-Mures, Romania; alina.muresan@umfst.ro (A.S.); cristiana-manuela.cojocaru@umfst.ro (C.M.C.); 2 Department of Ethics and Social Science, George Emil Palade University of Medicine, Pharmacy, Science and Technology, 540142 Targu-Mures, Romania; 3 Department of Psychology, West University of Timişoara, 4 Vasile Pâvan Boulevard, 300223 Timişoara, Romania; stefan.marian@e-uvt.ro; 4 Neurology Clinic I, Emergency Clinical County Hospital, 40136 Targu Mures, Romania; smaranda.maier@umfst.ro (S.M.); rodica.balasa@umfst.ro (R.B.); 5 Department of Neurology, University of Medicine, Pharmacy, Science and Technology, 540142 Targu Mures, Romania; 6 Faculty of Medicine, “Carol Davila” University of Medicine and Pharmacy, 050474 Bucharest, Romania; ovidiu.bajenaru@umfcd.ro; 7 National Institute of Gerontology and Geriatrics “Ana Aslan”, 11241 Bucharest, Romania

**Keywords:** multiple sclerosis, cognitive behavioral therapy, single-session intervention, depression, anxiety, negative automatic thoughts, dysfunctional attitudes, irrational beliefs, fatigue, perceived health

## Abstract

*Background:* Multiple sclerosis (MS) is one of the most debilitating neurodegenerative diseases in youth, significantly affecting all life domains. Therefore, a strong association between MS, depression and anxiety symptoms has been established. The objective of the present interventional one-group pretest–posttest study is to assess the feasibility of an online single-session intervention (SSI) based on a cognitive behavioral therapy protocol targeting depressive and anxiety symptoms, psychological mechanisms, fatigue and overall health status. *Methods:* In this pilot study, 31 patients with MS (M_age_ = 42 years, SD = 12.2) received the online CBT SSI. The impact of the intervention was assessed using validated instruments two weeks after the intervention and after a two-month follow-up period. *Results:* Statistically significant effects were observed for the reduction of depression, with B *=* −7.58, 95% CI (−12.84, −2.31) and *p* < 0.01, and anxiety, with B = −15.17, 95% CI (−18.31, −12.02) and *p* < 0.001, at post-test and follow-up screening. Additionally, positive outcomes were seen for irrational beliefs at post-test, with B = −25.86, 95% CI (−46.10, −5.61), along with negative automatic thoughts, with B = −4.47, 95% CI (−10.65, 1.71), which were preserved at follow-up. Despite the health status also improving, no significant changes were observed for dysfunctional attitudes and fatigue. *Conclusions:* This research proves that the online CBT SSI was efficient for decreasing mild to moderate symptoms of depression and anxiety and reducing the intensity of dysfunctional psychological mechanisms in our sample.

## 1. Introduction

Multiple sclerosis (MS) represents a neurodegenerative disease that impacts physical, cognitive, visual and sensory functioning. Neurological symptoms like numbness or tingling, weakness of the limbs, imbalance, spasticity and visual problems are frequently encountered. Along with these, patients often deal with urinary and intestinal dysfunctions, fatigue, cognitive impairment, and emotional distress. The prevalence of MS is estimated at 35.9 per 100,000, often occurring in comorbidity with emotional disorders such as depression and/or anxiety [1]. Sad mood, fear, irritability, psychomotor agitation, apathy, muscle tension and sleeping problems, accompanied by worries and ruminations related to the uncertainty of the disease’s progression, denote specific psychological difficulties encountered by MS patients. These may result in a wide range of negative consequences such as repeated hospitalizations, suicidal ideation, polypharmacy, unemployment and poor quality of interpersonal relationships. The vicious circle of dysfunctionality is further amplified through engagement in unhealthy coping strategies like avoidance, isolation, alcohol consumption and treatment non-adherence [2].

Based on its clinical manifestation, MS has several subtypes. Relapsing–remitting MS (RRMS) represents the most frequent subtype, characterized by acute attacks followed by complete recovery and no progression between relapses. The main symptoms consist of vision problems, neuropathy, unpleasant skin sensations, mental and physical tiredness, executive function deficits, grief, worry, fear of disease progression and anxious and depressive symptoms. Over time, the majority of patients diagnosed with RRMS will develop the secondary progressive MS (SPMS) subtype. This transition is explained by the debilitating progression of neurological symptoms with or without attacks. In addition to these common symptoms, patients experience significant walking difficulties, bladder and bowel problems, increased fatigue, cognitive impairment, depression and anxiety, sleeping problems and interpersonal difficulties. Primary progression MS (PPMS) is described as the progression of clinical manifestations with minimal improvements from the illness’s onset, representing the rarest subtype [3].

The co-occurrence of depression and anxiety is more frequent in patients with MS than in the general population [4]. The prevalence of clinical depressive and anxiety symptoms in MS patients ranges between 25 and 35% and between 21 and 35%, respectively [5,6], predominantly impacting the female gender [6]. Considering the subtypes of MS, the prevalence of depression and anxiety proved to be higher in progressive MS (PMS) (19.13% and 24.07%) as compared with the relapsing–remitting MS (RRMS) type (15.78% and 21.40%) [5]. 

The comorbidity between MS and major depressive disorder can be explained using the biopsychosocial model [7]. In this regard, neurobiological factors, such as brain injuries, cortical atrophies, and the progressive loss of gray matter in the limbic basal ganglion structures could be associated with depression [8,9]. A correlation between depressive symptoms and the treatment of MS with interferon beta (INFβ), such as INFβ-1a or INFβ-1b, has also been established [10]. Due to its immuno-modulatory properties, INFβ was the first disease modifying therapy used for treating RRMS with excellent results in reducing relapses and brain lesions, while also slowing down the disability [11]. Throughout the disease’s course, the occurrence of depression and anxiety could also be influenced by several social factors including age, education, marital and employment status, perceived limited social support and the quality of relationships, in addition to MS-specific symptoms and progression [12]. Specifically, the clinical picture characterized by fatigue and depression as main symptoms that are often interacting make it difficult to differentiate between these two conditions [13]. The most frequently cited psychological determinants of depression are learned helplessness, self-esteem, cognitive distortions, attributional style, negative affective memory errors, dysfunctional attitudes and high stress levels [14,15]. Moreover, in correlation with depression, the duration of disability and prolonged illness are the main factors associated with suicidal ideation and suicidal attempts, which occur with a higher frequency in MS patients than the general population [16]. As far as it is known, there is an overlap between MS symptoms and depression and/or anxiety [6]. In these circumstances, underdiagnosed depression and/or anxiety may reduce treatment compliance, worsening symptoms and disability and negatively impacting the general health status and quality of life [17,18]. 

The efficiency of different psychological interventions for reducing depressive and anxiety disorders was the subject of many studies conducted in this area. Therefore, alongside pharmacological treatments [19], psychological interventions like psychoeducation, supportive listening, counseling, cognitive behavioral therapies, mindfulness-based cognitive therapy, acceptance and commitment therapy and internet-based problem-solving therapy are recommended due to their effectiveness in the treatment of depression and anxiety symptoms associated with MS [20,21]. Internet-based cognitive behavioral therapies (CBTs) are among the most effective psychological interventions, presenting medium treatment effects on the reduction of psychopathology, relying on protocols between 5 and 12 sessions or more [22]. Cognitive behavioral therapy (CBT) represents a type of talking therapy developed by Aron T. Beck in the 1960s as a structured, collaborative, shot-term and present-focused approach for helping clients manage their problems by changing their unhelpful patterns of thinking and behaving. Since then, this therapy has been adapted to various disorders and specific populations through changing some of its characteristics, except for the theoretical model [23]. Hence, over time, cognitive behavioral interventions proved to be effective in reducing depression and anxiety symptoms linked to medical conditions [24,25,26].

Beck’s cognitive theory postulates that the emergence of negative emotions is not directly determined by external events but by the way in which that person thinks and interprets those events, through negative automatic thoughts (NATs). According to Beck’s cognitive theory, NATs represent evaluative patterns of thinking (e.g., “I am no good”; “My future is hopeless”) mostly occurring at a pre-conscious level, characterized by reality distortion and rigidity, which are directly responsible for the occurrence of dysfunctional emotions and unhelpful behaviors [23]. In conjunction with NATs, dysfunctional attitudes, described as cognitive distortions and profound beliefs about the self and the world (e.g., “Asking for help is a sign of weakness”; “The world is a dangerous place”), are maladaptive psychological mechanisms that predict clinical symptoms of depression and anxiety [27]. Likewise, irrational beliefs refer to a set of cognitive distortions and assumptions accountable for an inappropriate sense of reality, leading to dysfunctional negative emotions. These are described as “should” statements (e.g., “I must feel better”), personalization (e.g., ”Failing to keep my job is my fault”), dichotomous thinking (e.g., ”I am a failure because I ‘ve got sick”), overgeneralization (e.g., “All people are bad”). Therefore, these beliefs are divided into the following subtypes: demandingness, awfulizing/catastrophizing, self-downing/global evaluation and low frustration tolerance [28]. In this regard, CBT uses a series of specific techniques for helping patients develop an adaptive response for different stressful life situations [23]. Cognitive techniques like Socratic questions, cognitive restructuring, behavioral experiments, and behavioral techniques (behavioral activation, exposure, relaxation, problem solving) are used to increase one’s self-efficacy, control and functional attitude.

To date, a series of observational studies have identified positive correlations between depression, anxiety and dysfunctional psychological mechanisms in patients with MS [15,29]. However, there is insufficient evidence regarding the effect of CBT interventions on these dysfunctional psychological mechanisms.

Despite their positive outcomes on the mental health status of MS patients, CBT interventions have also presented shortcomings resulting in problematic accessibility or increased dropout rates in face-to-face interventions, given the difficulties related to mobility issues and other specific MS symptoms. These limitations have led to the development of several computerized CBT programs to facilitate the access of MS patients to psychological treatments [30,31]. However, following a generalized psychological program is often challenging for MS patients, since it is not developed to respond to their needs resulting from the interaction between disease symptoms and psychosocial factors. Also, the motivation to complete computerized modules is typically lower due to the therapist’s absence throughout the changing process. Thus, dropout rates are typically higher in computerized CBT programs in comparison with face-to-face CBT sessions [32]. 

Ultra-brief psychological interventions represent an alternative to full-length treatments, compacting core CBT techniques in up to five sessions, aiming to maximize efficiency, while also reducing the treatment duration. A typical session lasts between 15 and 45 min, involving a partially guided or unguided format [33]. A recent randomized controlled trial demonstrated that a guided ultra-brief treatment for depression and anxiety was equivalent to a standard-length treatment, leading to a significant decrease in depressive and anxiety symptoms at both post-test and 9-week follow-up assessments. In this study, the ultra-brief intervention consisted of an online lesson that could be accessed by participants throughout one month [34]. Single-session interventions (SSIs) are a specific type of well-structured ultra-brief treatment consisting of a unique session for developing the necessary cognitive, emotional and behavioral skills to manage psychological difficulties [35]. In a meta-analysis, Odgers et al. (2022) compared the efficiency of a single session to multi-session exposure for specific phobias, concluding that both approaches were equally effective. More than that, single-session exposure was found to be more time- and cost-effective [36]. Therefore, CBT SSIs could serve as an alternative to classical and computerized CBT programs for treating depression and anxiety in MS, facilitating treatment access especially for those individuals who face serious mobility difficulties or live in areas with poor availability of mental health services, such as rural places. For example, at a 3-month assessment, an SSI using a group CBT program for pain management had comparable outcomes with an eight-session CBT intervention for reducing the intensity of pain catastrophizing, as well as depression and anxiety symptoms in patients with chronic pain conditions [37]. 

Attempts to identify the best approaches for improving physical and mental health, as well as overall quality of life, are ongoing in research on the effectiveness of psychological interventions for MS patients. In this context, the main goal of the present study is to test the effectiveness and feasibility of an online CBT SSI tailored to MS patients. In contrast to the common ultra-brief approach, the particularities of this intervention consist in the higher length of the session and permanent therapist guidance. The first hypothesis states that there are positive correlations between depression, anxiety and dysfunctional psychological mechanisms. The second hypothesis is that the CBT SSI would be associated with a substantial decrease in depression and anxiety. Moreover, there is a scarcity of studies exploring the effects of CBT beyond symptoms and focusing on the dysfunctional psychological mechanisms involved in the onset of depression and anxiety in patients with MS. For this reason, the third hypothesis of this study is that the CBT SSI would decrease irrational beliefs, NATs and dysfunctional attitudes as main dysfunctional psychological mechanisms. In addition, based on previous findings indicating a strong correlation between psychopathology and core MS symptoms, the fourth hypothesis of our research is that the CBT SSI would improve fatigue levels and perceived health status. 

As far as we know, this is the first study investigating the impact of a psychological intervention on the reduction in dysfunctional psychological mechanisms in MS patients, reinforcing the role of these processes into the CBT framework.

## 2. Materials and Methods

### 2.1. Participants

In the present interventional one-group pretest–posttest study, we calculated the sample size by using G-Power software version 3.1 and performing an a priori analysis for repeated-measures *t* tests (matched pairs), at an effect size set to 0.5, power at 0.8 and alpha error likelihood of 0.05. The resulting output involved a total sample size of 34 participants, at a critical t = 2.03. Participants enrolled in the present study were selected from the database of the Neurological Clinic of the County Emergency Clinical Hospital in Targu Mures. All participants have been diagnosed with MS by a neurologist and were undergoing a disease modifying therapy (DMT). The study was carried out between November 2021 and October 2023, under the ethical approval issued by the Ethics Commission of Scientific Research of George Emil Palade University of Medicine, Pharmacy, Science and Technology from Targu Mures, number 1446, from 22 July 2021. The first phase of the study involved the screening of the participants regarding the presence of depression and/or anxiety using the Structured Clinical Interview for the DSM-5 Clinician Version (SCID-5-CV) [38]. The inclusion criteria were as follows: (1) a final diagnosis of MS according to McDonald criteria, (2) the presence of depression and/or anxiety according to the SCID-5-CV, (3) age equal or over 18 years old, (4) disability equal or lower than 7 as measured with the Expanded Disability Status Scale (EDSS), (5) minimum digital skills and (6) knowledge of the Romanian language. The exclusion criteria were as follows: (1) the diagnosis of other neurological diseases, (2) the presence of psychotic symptoms or schizophrenia, (3) moderate and severe cognitive impairment as evaluated using the Montreal Cognitive Assessment (MoCA) [39], (4) Interferon-β treatment, (5) any changes in medical treatment consisting of neurological and psychiatric medication or starting new medical treatment during the study and (6) engagement in another psychological treatment at the moment of inclusion. From a total number of 123 participants, 92 participants were excluded for failing to meet the criteria or refusing to engage in the next steps of the research and 4 participants dropped out during different stages of the study.

### 2.2. Measures

The Beck Depression Inventory II (BDI-II) [40] is an instrument that assesses the intensity of depressive symptoms. The questionnaire is a self-assessment tool containing 21 items that target the psychological and somatic symptoms of a major depressive episode. Each item is rated on a scale from 0 = low intensity to 3 = high intensity, summing up a maximum score of 63 points. The total score is interpreted according to the following cut-offs: 0–9, minimum depression; 10–18, moderate depression; 19–29, major depression; 30–36, severe depression. From a psychometric point of view, the questionnaire has very good internal consistency, reflected by a Cronbach’s alpha value of 0.91 [40]. The instrument was adapted for the Romanian population, presenting a Cronbach’s alpha level of 0.90 [41]. This instrument was used for assessing depression in MS patients in different settings, including online [22].

The Hamilton rating scale for anxiety (HARS) [42] is a clinical interview with 14 items that evaluate anxiety severity. The items assess both psychic and somatic anxiety. Each item is scored by the clinician using a Likert scale starting from 0 = lack of anxiety to 4 = severe anxiety, with a maximum total score of 56, where a score over 20 is considered the cut-off for clinically intense anxiety. The scale has high reliability and good internal consistency, indicating a Cronbach’s alpha level of 0.92 [43]. The scale was adapted for the Romanian population, obtaining an inter-rated concordance of 0.84 [44]. The interview was used to evaluate anxiety symptoms in MS patients [45] and proved to be more effective than self-rated questionnaires when applied in an online setting [46].

The Automatic Thoughts Questionnaire (ATQ) [47] is a 15-item self-report questionnaire that measures common negative self-statements or cognitions. Each item states a single thought, such as “Nothing feels good anymore” or “I’m a failure”. The frequency of each cognition is rated using a 5-point Likert scale starting from 1 = not at all to 5 = all the time. The total score is the sum of all ratings. The scale has been adapted for the Romanian population, demonstrating excellent internal consistency with a Cronbach’s alpha coefficient of 0.92 [48]. These psychometric properties were preserved in the MS population [29]. Also, the scale has been used for evaluating negative thinking in online assessments [49].

The Dysfunctional Attitudes Scale -A (DAS-A) [50] is a 40-item self-report measure of cognitive distortions and dysfunctional beliefs. The A form was applied in this study, consisting of assumptions like the following: ”If other people know what you are really like, they will think less of you” or “If I do not do as well as other people, it means I am an inferior human being”. Responses are rated on a 7-point Likert scale from 1 = totally disagree to 7 = totally agree. The total score is a summary of all individual ratings, encompassing several items that are reversely scored. The questionnaire has been adapted for the Romanian adult population, indicating high reliability, with an alpha coefficient of 0.86 [51]. This self-report scale was used to assess dysfunctional beliefs in MS patients by Guner et al. [15]. The DAS-A demonstrated its validity for establishing the relation be-tween dysfunctional beliefs and depression [52].

The Attitudes and Belief Scale Second Edition (ABS-II) [53] is a 72-item self-reported scale that measures irrational and rational beliefs as the central constructs of rational emotive behavioral therapy. Items are distributed in a matrix based on three factors: cognitive processes (demandingness—DEM, global evaluation—GE, low frustration tolerance—LFT, awfulizing—AWF), context (approval, achievement and comfort) and phrasing (irrationality/rationality). Each item is rated on a 5-point Likert scale starting from 0 = strongly disagree and ending with 5 = strongly agree (e.g., “I can’t stand not being liked by certain people”, and “I can’t stand the idea that they might not like me”). High scores indicate increased levels of irrationality. The scale was adapted for the Romanian population with an alpha Cronbach’s of 0.87 for the total score [54]. As far as we know, this is the first time it has been applied in MS patients. The online application of the scale was conducted only in the general population [55].

The Chalder Fatigue Scale (CFS) [56] includes 11 items that assess physical and psychic symptoms of fatigue. Each item can be scored using a 4-point Likert scale from 0 to 3, where 0 = better than usual, 1 = no worse than usual, 2 = worse than usual and 3 = much worse than usual. The higher the total score, the greater the fatigue [56]. The scale reliability was observed in several online investigations involving MS patients [22].

The EQ-5D-3L is a standardized self-reported measure of health comprising 5 dimensions: mobility, self-care, usual activities, pain/discomfort and anxiety/depression [57]. The evaluation of each dimension comprises a three-level assessment, using a Likert scale from 1 to 3, where 1 = no problems, 2 = moderate problems and 3 = severe problems. The dimension score is represented by an index composed of 5 digits (e.g., 11,111, 21,212) representing the 3 levels of evaluation of the domain, providing an insight into the patient’s health status. The instrument also incorporates a visual analogue scale (VAS) that captures the evaluation of the patient’s present health, reported on a scale from 0, representing the worst health status, to 100, reflecting the best health status [57]. Several studies have used this instrument to measure the health status of MS patients within an online environment [22].

### 2.3. Design and Procedure

**Figure 1:**
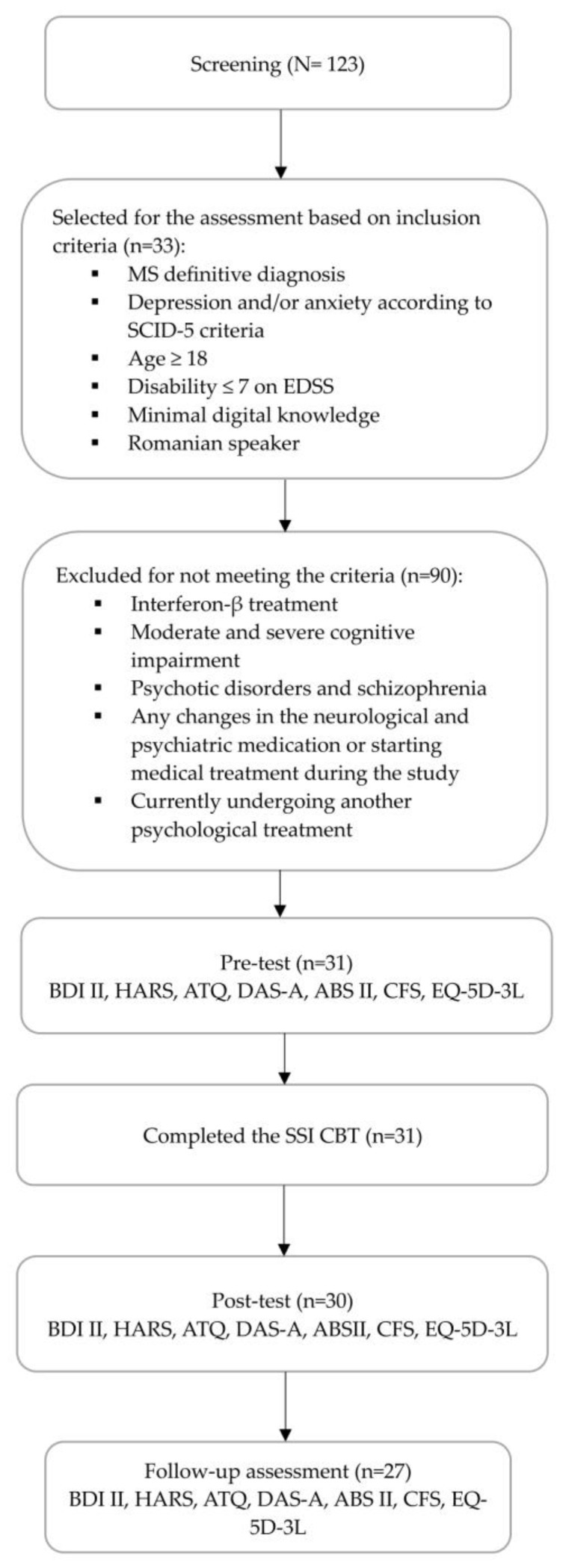
The flow diagram of the study procedure.

This study employed a one-group pretest–posttest design for evaluating an online CBT SSI, including 31 MS patients who presented a disability score ≤ 7 as measured by the Expanded Disability Status Scale (EDSS), as well as an average cognitive impairment of 25.9 (SD = 2.05). Figure 1 summarizes the sample flow of participants. The screening applied the SCID-5 for assessing the presence of depression and/or anxiety symptoms in MS patients. Participants who met the criteria for at least one or both disorders were enrolled in the study. At the time of enrollment, from the entire study sample (N = 31), seven patients were currently receiving treatment with antidepressant medication. Before the intervention was conducted, depression and anxiety intensity were assessed using the BDI II and HARS, along with the levels of fatigue and perceived health with CFS and EQ-5D-3L, respectively. For evaluating psychological mechanisms, the ATQ, DAS-A and ABS II self-reports were used. All clinical instruments were applied two weeks after the intervention and after 2 months during a follow-up assessment. The assessment was conducted online for the self-reported clinical instruments. Also, the interview for the assessment of anxiety intensity was carried out by a clinical psychologist, involving an online meeting platform.

The present protocol was elaborated and used for the first time in this study. The intervention was developed based on Beck’s CBT protocol, consisting of psychoeducation, clinical case conceptualization, cognitive modification, behavioral and emotional strategies, an action plan and relapse prevention [23]. The present protocol involves an SSI, conducted online by a licensed cognitive behavioral psychotherapist. The 90 min individual session followed the main stages of an SSI: introduction, middle and conclusion [58]. Thus, the first phase included a short psychoeducation session on functional and dysfunctional emotions and a description of the cognitive model [59]. In the second phase, the clinical case conceptualization based on Beck’s simple cognitive model was introduced [23]. The third phase consisted of the identification of NATs in relation to the disease or to the consequences of the disease for personal, social and professional areas of functioning. The core strategies of the cognitive intervention were the evaluation and restructuring of NATs, using pro–con and cost–benefit analyses. Also, mindfulness of breath was another technique used for NAT management [60]. This technique teaches patients to get in contact with their negative thoughts by observing and accepting their presence, without analyzing them, engaging in the present moment in this way [23,61]. In the fourth phase, behavioral activation was introduced to increase the frequency of pleasant, meaningful activities derived from the patients’ aspirations/life values. Moreover, the Jacobson progressive muscle relaxation technique was practiced to counteract the uncomfortable physiological reactions defining anxiety disorders [62]. The last part of the SSI was dedicated to sleep hygiene. At the end of the session, a behavioral action plan was established, including activity scheduling. In this regard, within a collaborative therapeutic framework, patients received proper information on the application of cognitive and behavioral techniques practiced during the session for practicing these skills during the following period, until the follow-up assessment. This action plan included the following: (1) emotional regulation techniques (adapted from Barlow et al.) [59]; (2) a thought record worksheet [23]; (3) mindfulness techniques [60]; (4) a relaxation technique (Jacobson progressive muscle relaxation) [62]; (5) the CBT-I-Sleep hygiene mini guide, adapted from Manber et al. [63]. This action plan was personalized to the specific psychological problem of each patient, implementing a daily schedule until the 2-month follow-up. Throughout the entire period of this study, patients had the possibility to contact the therapist if needed. During the entire period of the study, no patient contacted the psychotherapist. The psychotherapist interacted with patients exclusively during the SSI. In terms of professional background, the therapist was a clinical psychologist and a trained CBT psychotherapist, certified by the Romanian Association of Behavioral and Cognitive Psychotherapy, with at least 17 years of experience, practicing within the public health care system and private practice. Throughout the elaboration of the protocol, the therapist was guided by a senior CBT psychotherapist certified at the Beck Institute from Philadelphia, USA.

### 2.4. Statistical Analysis

Data analysis was carried out using R, version 4.3.2 [64], and IBM SPSS software, version 29.0.1. Pearson’s correlations were performed to investigate and establish the relations between different variables. To evaluate the effect of the intervention, we started with a simple comparison using paired-samples *t* tests between the pre-test and at post-test scores for each outcome variable. We performed this analysis using the package rstatix [65], which was also used to obtain within-subjects Cohen’s d (Cohen’s dz) as an estimate of effect size. For a more in-depth evaluation of the intervention’s effect, we continued the analysis by estimating a mixed-effects model for each dependent variable using the package lme4 [66], which allows for the control of covariates and the handling of missing data. In the analysis we tested for a significant change at post-test screening and controlled for the covariates MoCA, EDSS, sex, age and psychiatric treatment (yes or no). This approach was also chosen for the ability to handle missing data using maximum likelihood estimation with all available data in long format, preventing listwise deletion [67]. Additionally, we used the package *lmerTest* to obtain *p* values for the fixed effects [68]. The models included time, the mentioned covariates, the interactions between time and covariates as predictors and a random intercept for the subject. The same analytic strategy was applied to test the effects during the follow-up period. Time was coded with 0 = pre-intervention and 1 = post-intervention (or follow- up) so that the intercept reflects the estimated mean before the intervention and the beta for time reflects the estimated change post intervention (or at follow-up). Numerical covariates were grand mean centered. Gender was coded with 0 = female and 1 = male. To assess univariate normality, we calculated skewness and kurtosis for each time-point and assessed whether skewness for each variable is in the −2–+2 interval and kurtosis in the −7–+7 interval. Assumptions of the homoscedasticity of residuals, normality of residuals, and outliers were inspected visually using the *plot_model* function from the package *sjPlot* [69]. The sjPlot package was also used to obtain marginal and conditional r squared values using the function tab_model. Additionally, we inspected scatterplots of residuals and the outcome variable. Because the model involved covariates, we also estimated marginal means using the package *emmeans* [70].

## 3. Results

The sample included 31 MS patients with a mean age of 42.3 years (SD = 12.2). The percentage of females was 74.20% and the large majority, 90.30%, were diagnosed with RRMS. In the sample, 38.70% of the patients had minimal depression, 22,58% had mild depression, 25.80% scored for moderate depression and 12.90% were severely depressed. As for the intensity of anxiety symptoms, 9.67% presented mild intensity, 41.93% were moderately anxious and 48.39% of scores indicated moderate to severe levels of anxiety. Additionally, 41% of the patients were already retired, while 38.70% declared that they were employed. More than half of the patients were married. The sociodemographic and medical characteristics of participants included in this study are presented in Table 1.

Table 2 presents the results of the correlation between variables indicating clinical symptoms and dysfunctional psychological mechanisms during the pre-test, post-test and follow-up assessments. All variables presented a normal distribution. Statistically significant positive correlations between symptom variables and all investigated psychological mechanisms were observed during the pre-test and follow-up assessments. Despite this, the correlations between anxiety and ABS II-DEM, ABS II-ach and DAS-A, as well as between depression and ABS II-DEM, ABS II- AWF and ABS II-ach were not statistically significant during post-test screening.

Table 3 illustrates the descriptive statistics and pairwise comparisons for all the variables of the pre-test mean with the means measured during post-test and follow-up screening. All variables were normally distributed at each of the three timepoints, with values for skewness falling in the −2–+2 interval and in the −7–+7 interval for kurtosis.

Table 4 presents the results from the post-test assessment. The table includes unstandardized regression estimates, confidence intervals, variance parameters for random effects and the proportion of variance explained by fixed effects (marginal r2) and by both random and fixed effects (conditional r2). The presented coefficients reflect results when controlling for covariates. We obtained a significant effect of time for all variables in the expected direction. Regarding the first objective, after the CBT SSI was implemented in our study, depressive B = −7.58, 95%CI (−12.84, −2.31), *p* < 0.01 and anxiety symptoms B = −15.17 [−18.31, −12.02], with *p* < 0.001 reduced from the pre-test to post-test period.

Table 5 presents the results from follow-up screening. From the pre-test to the follow-up period, both depression, with B = −8.08, 95%CI [−13.60, −2.56] and *p* < 0.001, and anxiety symptoms, with B = −19.45, 95%CI [−22.88, −16.03] and *p* < 0.001, were reduced, reaching the same statistical significance. The effects measured during the follow-up assessment were not significant for the variables ABS-DEM, ABS-SDG, ABS-LFT, ABS-re, ABS-ap, ABS-IR, DAS-A, ABS-R, DAS-A and CFS. The effect of time remained significant for all the other measures.

Table 6 presents the estimated marginal means. These results reflect the mean and the standard error estimated at the three timepoints when controlling for covariates.

Some effects of covariates and interactions between covariates and time were significant. These results are presented in Table 7. For the purpose of conciseness, we report only the effects of covariates that were significant.

## 4. Discussion

The results of our study showed that an SSI based on CBT was efficient for improving the subjective health status of MS patients with mild to moderate anxiety and depression symptoms. These results are in accordance with previous studies highlighting an overall improvement in functional outcomes in chronic medical conditions following an SSI using a CBT framework [71]. Also, this emphasizes that a brief intervention targeting the main difficulties of MS patients might resemble the effects of full-length CBT protocols in this specific population.

Regarding the first hypothesis, a significant correlation between depression, anxiety, NATs, irrational beliefs and dysfunctional attitudes was observed for all measurements. In other words, the reduction in dysfunctional psychological mechanisms is directly linked to a decrease in depressive and anxiety symptoms. These findings are consistent with previous studies that have identified the same correspondence between emotional distress and dysfunctional psychological mechanisms [15,29,72,73]. Moreover, some of these studies demonstrated the moderating effects of dysfunctional attitudes in the relation between negative life events and depression, as well as the mediating effect of NATs on depressive and anxiety symptoms [72]. Likewise, Batmaz (2015) highlighted the predictive role of dysfunctional attitudes in the recurrence of MDD episodes [74]. During post-test screening, the positive correlations between dysfunctional attitudes, NATs and depressive symptoms were preserved. We consider this to be justified through the lens of previous investigations that underlined the central role of NATs and dysfunctional attitudes in the onset of depression [55]. Also, secondary irrational beliefs like self-downing/global evaluation and low frustration tolerance were particularly associated with depressive thoughts [55,73]; therefore, the expected relationship with depressive symptoms was maintained. Furthermore, the interaction between NATs and anxiety during the post-test assessment was maintained, as expected. Indeed, these psychological mechanisms were found to mediate anxiety symptoms. At the same time, the predictive role of dysfunctional attitudes was not significant for anxiety [75,76]. On the other hand, awfulizing and low frustration tolerance as irrational beliefs were associated with anxiety in particular [55]. We believe this justifies the maintenance of correlations. In contrast, we consider that the absence of statistically significant correlations between demandingness, irrational beliefs, depression and anxiety could be understood through the nature of anxiety and depression in MS, which should be explored in further studies. Nevertheless, the correlations obtained between the follow-up measurements could be explained by the lasting effects of practicing the cognitive and behavioral techniques established in the action plan during the SSI, along with other contextual factors that were not assessed. Therefore, we assume that the routine evaluation of dysfunctional psychological mechanisms, along with the assessment of clinical symptoms, would be beneficial for preventing the onset of clinical distress. 

As for the second hypothesis, the CBT SSI implemented in our study was efficient in reducing depressive and anxiety symptoms. These results are aligned with the literature on the benefits of brief CBT interventions for alleviating depression and anxiety symptoms in chronic diseases [37,77]. Moreover, this outcome was preserved from the post-test assessment to the follow-up screening, in terms of both depression and anxiety. In the same way, past analyses underlined the lasting positive effects of short CBT interventions for psychological adjustment [37,78]. A strength of our investigation was the use of both self-reports and clinician-rated instruments for the assessment of psychopathology, indicating similar results at both temporal points. In our opinion, the observed benefits could be related to the integrative nature of the intervention that combined cognitive and behavioral techniques, adapting them to the specificity of MS. Also, using psychoeducation is known to improve the clinical picture in patients with chronic medical conditions. 

The third hypothesis of our study regarding the involvement of dysfunctional psychological mechanisms in depression and/or anxiety was only partially confirmed. In this respect, we noticed that irrational beliefs were reduced after the CBT SSI. More specifically, the level of irrational beliefs decreased when measured during the both post-test and follow-up screenings. These results can be explained through the fact that the CBT SSI only addressed “cold cognitions”, which are generally considered a type of surface-level thinking [79].

Interestingly, although the intensity of NATs remained unmodified in terms of the post-test screening, it reduced significantly during the follow-up period. We consider that this discrepancy related to the intensity of NATs between the two assessments could be the result of the patients’ willingness to follow the personalized action plan consisting of cognitive, behavioral and mindfulness techniques until the follow-up assessment.

Another explanation could be attributed to NATs’ description as “hot cognitions” (i.e., evaluative, less conscious, unmotivating and mostly negative thoughts) that trigger and exacerbate emotional, behavioral and physiological dysfunctional reactions. Therefore, the identification and modification of NATs’ contents and significations require a longer duration for the development of a more adaptative behavioral response to internal and external events [23,80]. Our results are consistent with the study of Troeung et al. (2014), observing a statistically significant reduction in negative thinking related to depression and anxiety disorders in patients with Parkinson’s disease after adjusting a CBT protocol to the specific nature of this condition [81]. Another possible explanation is the integrative nature of the protocol, which incorporated mindfulness and CBT techniques guiding patients to become more self-efficient and self-controlled in accepting and tolerating negative emotions in order to cope with the disease’s course [82]. Moreover, among the therapeutic processes involved throughout the session for alleviating symptom intensity, we have mentioned cognitive restructuring, emotion regulation, collaboration and the action plan [83]. Likewise, studies on the mechanisms/processes of action in CBT have outlined the role of the cognitive dimension for facilitating change in these approaches [84,85]. At the same time, the NAT level was influenced by the interactive effects between the intervention and degree of cognitive impairment. These results reinforce the idea that the negative thinking styles found in depression and anxiety positively correlate with memory, information processing and executive functioning deficits in MS [86].

Dysfunctional attitudes are the only mechanisms that did not decrease after the CBT SSI. We consider that challenging dysfunctional attitudes would require multiple psychotherapeutic sessions, together with other specific cognitive behavioral strategies, since they constitute the core beliefs of the cognitive triad.

Concerning the fourth hypothesis, the positive effect of the intervention on health alone has been confirmed. This finding stresses the role of cognitive and behavioral techniques for lifestyle change, facilitating physical and mental wellbeing in MS patients. In terms of perceived health, several quality-of-life components increased, including mobility, engaging in daily activities, self-care, lack of pain and psychological well-being. This is in accordance with other studies that proved the importance of CBT in the clinical management of long-term medical conditions [87]. In line with previous investigations, disability scores predicted the intervention’s impact on aspects related to quality of life in patients with MS [88].

In contrast to findings regarding previous CBT protocols that were associated with a decrease in fatigue levels as a result of emotional distress alleviation, our intervention did not achieve the same outcome. An explanation could be related to the shortness of our intervention. For example, a meta-analysis conducted by Van der Akker et al. (2016) showed that although the level of fatigue improved in MS patients who pursued a CBT intervention consisting of four to eight sessions, the fatigue level started to increase shortly after the end of the psychotherapeutic process [89]. Another argument relies on the multifactorial origin of fatigue in MS, which points to the necessity of diverse interventional approaches [90]. 

Moreover, the findings of the present study could be influenced by the characteristics of the intervention provider. Possibly, expertise in the field of clinical work could facilitate protocol adjustment to aid the patients’ response to the psychological treatment. As a future direction, upcoming investigations could test the impact of the same protocol applied by therapists with different degrees of expertise.

Nonetheless, beyond the outcomes, this pilot study involves some limitations. One of the main constraints was related to the lack of a control group, which hinders the interpretability of the positive results obtained in this research. For this reason, comparisons between the effects of different protocol components and other confounds could not be performed. Another limitation is the small sample size, which does not allow the extrapolation of our findings to the entire population in the target group. Additionally, these results can be attributed only to the specific patient group included in our study, specifically to the RRMS type of MS, which represented the majority of our sample. Also, due to specific MS symptomatology, the length of the protocol could be demanding within a digital environment. Therefore, future studies could test the protocol in a face-to-face format. A limitation of the present protocol is that the cognitive strategies focused predominately on NATs and less on dysfunctional attitudes, given the brief duration of the intervention. Given the impossibility to address higher-order dysfunctional cognitive mechanisms and exposure techniques that particularly target anxiety disorders in a unique session, we assume that the SSI is feasible only for mild to moderate depressive and anxiety symptoms, which were present in our group. An important direction for future research would be to assess the benefits of this SSI CBT including a passive and active control group within rigorously conducted randomized controlled trials. This would allow for the comparative evaluation of different change mechanisms as a function of different covariates, permitting the development of a more personalized approach. Moreover, a larger sample size would be required for extrapolating the positive outcome of the SSI CBT with greater confidence. In addition, more studies analyzing the role of each intervention component for patients with specific MS characteristics would be necessary. Likewise, a larger diversity of cognitive techniques could increase the effectiveness of the SSI.

### Clinical Implications

The protocol in the present study can be applied especially for MS patients with limited access to mental health services as a consequence of their socio-economic background and several demographic factors. Also, the use of brief CBT for reducing anxiety, depression and associated dysfunctional psychological mechanisms may promote compliance with medical treatments, ultimately improving the overall health status. At the same time, the action plan can serve as a useful tool for consolidating the skills acquired during the session. Furthermore, besides its application in ambulatory encounters, this digital intervention could be adapted for in-patient contexts, integrating this protocol within the standard treatment applied during hospitalization periods. A positive feature of the SSI is that it can be implemented by trained medical staff to improve the general care of MS patients as part of a holistic approach for preventing the development and exacerbation of psychopathology.

## 5. Conclusions 

This pilot study showed that the application of an online CBT SSI has an immediate and long-term impact on reducing depression and anxiety symptoms while also improving perceived health in patients with MS. Additionally, in conjunction with anxious and depressive symptoms, irrational beliefs and NATs decreased following this intervention. Therefore, a regular clinical evaluation of psychopathology is recommended in order to identify those patients who might benefit from this psychological intervention, emphasizing the importance of psychological well-being in the management of this disease. Nonetheless, future research could compare the benefits of the CBT SSI with other empirically validated psychological interventions in MS for establishing a more definitive efficiency.

## Figures and Tables

**Table 1:** Sociodemographic and medical characteristics.

	N	%
Sex		
Female	23	74.20%
Male	8	25.80%
Marital		
Divorced	3	9.70%
Married	21	67.70%
Single	7	22.60%
Area		
Rural	10	32.30%
Urban	21	67.70%
Education		
High School	11	35.50%
Higher	14	45.20%
Middle school	1	3.20%
Middle School	1	3.20%
Post high school	1	3.20%
Professional	3	9.70%
Occupation		
Employed	12	38.70%
Housewife	2	6.50%
Retired	13	41.90%
Student	4	12.90%
Diagnostic		
PPMS	2	6.50%
RRMS	28	90.30%
SPMS	1	3.20%
Disease Modifying Therapies		
Teriflunomide	7	22.60%
Ocrelizumab	17	54.80%
Glatiramer acetat	4	12.90%
Dimethyl furamate	3	9.70%
EDSS score		
0–1.5	1	3.22%
2–2.5	8	25.80%
3–3.5	15	48.38%
4–4.5	5	16.12%
5–5.5	2	6.45%
6–6.5	7	22.58%
7	1	3.22%
Psychiatric treatment		
No	24	77.40%
Yes	7	22.60%
Psychiatric disorder in antecedent		
Anxiety-depressive disorder	6	19.40%
Anxiety disorder	2	6.50%
Depressive disorder	3	9.70%
Generalized anxiety disorder	2	6.50%
None	18	58.10%
BDI-II		
Minimal	10	38.70%
Mild	7	22.58%
Moderate	8	25.80%
Severe	4	12.90%
HARS		
Mild	3	9.67%
Mild to moderate	13	41.93%
Moderate to severe	15	48.39%

Note: EDSS = Expanded Disability Status Scale, PPMS = primary progressive MS, RRMS = relapsing–remitting MS, SPMS = secondary progressive MS, BDI-II = Beck Depression Inventory and HARS = Hamilton Anxiety Rating Scale.

**Table 2:** Correlation between depression, anxiety and psychological mechanisms at all measurements.

	HARS			BDI		
	Pre-Test	Post-Test	Follow-Up	Pre-Test	Post-Test	Follow-Up
**HARS**	-	-	-	0.47 **	0.60 **	0.62 **
**BDI-II**	0.47 **	0.60 **	0.62 *	-	-	-
**ABS II-DEM**	0.40 *	0.30	0.55 **	0.49 **	0.27	0.42 *
**ABS II-SD/GE**	0.58 **	0.41 *	0.47 *	0.54 **	0.40 *	0.49 **
**ABS II-LFT**	0.56 **	42 *	0.44 *	0.57 **	0.50 **	0.44 *
**ABS II-AWF**	0.49 **	0.39 *	0.48 *	0.68 **	0.36	0.44 *
**ABS II-co**	0.50 **	0.44 *	0.53 **	0.62 **	44 *	0.42 *
**ABS II-ach**	0.57 **	0.27	0.42 *	0.60 **	0.32	0.46 *
**ABS II- app**	0.49 **	41 *	0.50 **	0.54 **	0.37 *	0.45 *
**ABS II**	0.55 **	0.39 *	0.52 **	0.61 **	0.39 *	0.47 *
**DAS-A**	0.47 **	0.26	0.69 **	0.53 **	47 **	0.45 *
**ATQ**	0.56 **	0.60 **	0.62 **	0.66 **	0.81 **	0.73 **

Note: ** *p* < 0.01, * *p* < 0.05; HARS = Hamilton anxiety rating scale, BDI-II = Beck Depression Inventory, ABS-DEM = ABS-II—demanding, ABS-SD/GE = ABS-II—self-downing/global evaluation, ABS-LFT = ABS-II—low frustration tolerance, ABS-AWF = ABS-II—awfulizing, ABS-co = ABS-II—comfort, ABS-ach = ABS-II—achievement, ABS-app = ABS-II—approval, ABS II = attitudes and beliefs scale, DAS-A = dysfunctional attitudes scale, ATQ = automatic thoughts questionnaire.

**Table 3:** Comparisons of the post-test and follow-up means with the pre-test means using the paired-sample *t* test.

Variable	Time	M	SD	skew	kurt	t	df	d_z_
**HARS**	Pre-test	24.1	5.97	0.11	−0.13			
	Post-test	9.9	6.37	0.57	0.21	11.42 ***	29	2.09
	Follow-up	5.07	5.74	1.10	−0.04	15.96 ***	26	3.07
**BDI-II**	Pre-test	18.26	10.41	1.05	0.95			
	Post-test	8.37	7.32	1.41	2.10	5 ***	29	0.91
	Follow-up	9.15	7.16	1.20	0.90	4.89 ***	26	0.94
**ABS-DEM**	Pre-test	25.77	13.7	0.17	−1.03			
	Post-test	20.1	14.05	0.28	−1.14	2.69 *	29	0.49
	Follow-up	22.11	12.31	0.08	−0.90	2.13	26	0.41
**ABS-SDG**	Pre-test	19.16	15.95	0.92	0.08			
	Post-test	15.63	12.44	0.42	−1.53	1.52	29	0.28
	Follow-up	14.96	11.19	0.38	−1.53	2.16	26	0.42
**ABS-LFT**	Pre-test	27.65	14.58	0.45	−0.21			
	Post-test	22.63	12.61	−0.18	−1.14	1.84	29	0.34
	Follow-up	21.96	13.82	0.13	−0.91	2.66 *	26	0.51
**ABS-AWF**	Pre-test	28.06	13.19	0.33	−0.65			
	Post-test	21.63	12.33	0.02	−1.48	3.26 **	29	0.59
	Follow-up	22.44	11.49	0.21	−1.01	3.99 **	26	0.77
**ABS-co**	Pre-test	35.39	17.47	0.49	−0.01			
	Post-test	28.27	16.01	−0.15	−1.59	2.44 *	29	0.45
	Follow-up	28.7	13.91	−0.18	−0.79	3.15 *	26	0.61
**ABS-ach**	Pre-test	35.74	18.97	0.72	0.79			
	Post-test	28.57	18.13	0.48	−0.38	2.56 *	29	0.47
	Follow-up	29.48	17.43	0.51	−0.27	2.51 *	26	0.48
**ABS-app**	Pre-test	29.48	19.57	0.39	−1.10			
	Post-test	23.3	17.24	0.19	−1.55	2.12	29	0.39
	Follow-up	23.3	17.5	0.22	−1.51	2.79 *	26	0.54
**ABS-IR**	Pre-test	62.71	30.02	0.16	−0.71			
	Post-test	51.73	28.83	0.03	−1.24	2.05	29	0.37
	Follow-up	51.93	28.93	0.37	−0.94	2.58 *	26	0.5
**ABS-R**	Pre-test	37.94	27.04	0.78	−0.13			
	Post-test	28.13	23.56	0.58	−1.06	2.51 *	29	0.46
	Follow-up	29.52	21.8	0.48	−1.05	2.98 *	26	0.57
**ABS II**	Pre-test	100.65	53.63	0.58	−0.22			
	Post-test	80.17	48.75	0.14	−1.54	2.5 *	29	0.46
	Follow-up	81.44	46.34	0.30	−1.03	2.99 *	26	0.58
**DAS-A**	Pre-test	126.55	44.79	0.98	0.65			
	Post-test	108.6	38.04	0.35	−1.13	2.43	29	0.44
	Follow-up	114	39.3	0.18	−1.28	1.9	26	0.37
**ATQ**	Pre-test	35.77	12.54	0.53	−0.42			
	Post-test	28.63	10.95	0.61	−0.42	2.9 *	29	0.53
	Follow-up	25.67	8.26	0.64	−0.47	4.81 ***	26	0.93
**CFS**	Pre-test	17.87	6.33	−0.25	−0.09			
	Post-test	11.77	6.35	0.34	−0.73	4.34 ***	29	0.79
	Follow-up	12.26	6.46	−0.19	−0.76	3.63 **	26	0.7
**EQ5D3L**	Pre-test	8.55	1.67	0.33	−0.76			
	Post-test	7.7	1.9	0.15	−1.07	5 ***	29	0.91
	Follow-up	7.74	1.89	0.13	−1.34	2.79 *	26	0.54
**EQ-VAS**	Pre-test	61.84	15.08	0.17	−1.10			
	Post-test	72.6	17.74	−0.46	−0.61	−4.04 **	29	−0.74
	Follow-up	72.78	17.23	−0.59	0.26	−3.41 **	26	−0.66

Note: *** *p* < 0.001, ** *p* < 0.01, * *p* < 0.05; N_pre_ = 31, N_post_ = 30; d_z_—Cohen’s d estimated based on standard deviation of pre-post differences; HARS = Hamilton anxiety rating scale, BDI = Beck Depression Inventory, ABS-DEM = ABS-II—demanding, ABS-SD/GE = ABS-II—self-downing/global evaluation, ABS-LFT = ABS-II—low frustration tolerance, ABS-AWF = ABS-II—awfulizing, ABS-co = ABS-II—comfort, ABS-ach = ABS-II—achievement, ABS-app = ABS-II—approval, ABS-IR = ABS-II—irrationality, ABS-R = ABS-II—rationality, ABS II = attitudes and beliefs scale, DAS-A = dysfunctional attitudes scale, ATQ = automatic thoughts questionnaire, CFS = Chalder Fatigue Scale, EQ-5D-3L = health state and EQ-VAS = health rated on a visual analogue scale.

**Table 4:** Results of mixed linear models for post-interventional effects.

		B	95% CI	σ^2^	τ_00_	ICC	r_m_^2^/r_c_^2^
**HARS**	Intercept	24.57 ***	21.65, 27.48	20.87	16.29	0.44	0.610/0.781
	Time	−15.17 ***	−18.31, −12.02				
**BDI-II**	Intercept	16.3 ***	11.73, 20.86	58.5	32.8	0.36	0.248/0.518
	Time	−7.58 **	−12.84, −2.31				
**ABS-DEM**	Intercept	26.08 ***	19.40, 32.75	69.65	125.41	0.64	0.158/0.699
	Time	−6.35 *	−12.12, −0.59				
**ABS-SDG**	Intercept	19.87 ***	12.93, 26.81	86.9	123.68	0.59	0.134/0.643
	Time	−6.54 *	−12.98, −0.10				
**ABS-LFT**	Intercept	29.64 ***	23.21, 36.07	73.08	107.9	0.6	0.192/0.674
	Time	−5.31	−11.21, 0.60				
**ABS-AWF**	Intercept	28.97 ***	22.62, 35.33	52.33	124.53	0.7	0.127/0.742
	Time	−7.66 **	−12.66, −2.65				
**ABS-co**	Intercept	38.44 ***	30.27, 46.61	115.55	176.63	0.6	0.144/0.661
	Time	−10.78 **	−18.20, −3.35				
**ABS-ach**	Intercept	36.4 ***	27.70, 45.10	96.23	235.05	0.71	0.196/0.766
	Time	−7.09 *	−13.87, −0.30				
**ABS-app**	Intercept	29.7 ***	20.69, 38.71	104.06	251.3	0.71	0.127/0.744
	Time	−8.03 *	−15.09, −0.98				
**ABS-IR**	Intercept	67.87 ***	54.05, 81.68	337.44	498.18	0.6	0.192/0.674
	Time	−12.42	−25.11, 0.27				
**ABS-R**	Intercept	36.69 ***	24.32, 49.05	234.51	434.69	0.65	0.138/0.698
	Time	−13.52 *	−24.10, −2.93				
**ABS II**	Intercept	104.56 ***	79.82, 129.30	857.31	1821.87	0.68	0.154/0.729
	Time	−25.86*	−46.10, −5.61				
**DAS-A**	Intercept	122.77 ***	103.18, 142.35	701.67	977.58	0.58	0.192/0.663
	Time	−12.07	−30.37, 6.22				
**ATQ**	Intercept	34.68 ***	28.75, 40.62	80.21	74.08	0.48	0.131/0.548
	Time	−4.47	−10.65, 1.71				
**CFS**	Intercept	15.9 ***	13.02, 18.79	25.43	10.97	0.3	0.346/0.543
	Time	−3.37	−6.84, 0.10				
**EQ5D3L**	Intercept	8.39 ***	7.79, 8.99	0.4	1.17	0.75	0.563/0.889
	Time	−0.77 **	−1.20, −0.33				
**EQ-VAS**	Intercept	61.41 ***	54.47, 68.34	113.95	96.38	0.46	0.368/0.658
	Time	11.95 **	4.59, 19.31				

Note: *** *p* < 0.001, ** *p* < 0.01, * *p* < 0.05; N_pre_ = 31, N_post_ = 30; N_follow-up_ = 27; B—unstandardized regression estimate, 95% CI—95% confidence interval, σ^2^—residual variance, τ00—variance explained by random effects (subjects), ICC—intraclass correlation, r*_m_^2^*—marginal r squared (variance explained by fixed effects only), r*_c_^2^—*conditional r squared (variance explained by fixed and random effects), HARS = Hamilton anxiety rating scale, BDI = Beck Depression Inventory, ABS-DEM = ABS-II—demanding, ABS-SD/GE = ABS-II—self-downing/global evaluation, ABS-LFT = ABS-II—low frustration tolerance, ABS-AWF = ABS-II—awfulizing, ABS-co = ABS-II—comfort, ABS-ach = ABS-II—achievement, ABS-app = ABS-II—approval, ABS-IR = ABS-II—irrationality, ABS-R = ABS-II—rationality, ABS II = attitudes and beliefs scale, DAS-A = dysfunctional attitudes scale, ATQ = automatic thoughts questionnaire, CFS = Chalder Fatigue Scale, EQ-5D-3L = health state and EQ-VAS = health rated on a visual analogue scale.

**Table 5:** Mixed linear models of results for follow-up effects.

		B	95% CI	σ^2^	τ_00_	ICC	r_m_^2^/r_c_^2^
**HARS**	Intercept	24.57 ***	21.69, 27.45	22.23	13.98	0.39	0.731/0.835
	Time	−19.45 ***	−22.88, −16.03				
**BDI-II**	Intercept	16.29 ***	11.77, 20.82	57.89	31.28	0.35	0.256/0.517
	Time	−8.08 **	−13.60, −2.56				
**ABS-DEM**	Intercept	26.09 ***	19.78, 32.41	71.9	102.06	0.59	0.151/0.649
	Time	−2.8	−9.02, 3.42				
**ABS-SDG**	Intercept	19.87 ***	13.14, 26.59	82.86	114.55	0.58	0.151/0.644
	Time	−6.44	−13.12, 0.23				
**ABS-LFT**	Intercept	29.65 ***	22.92, 36.38	82.43	115.02	0.58	0.207/0.669
	Time	−5.09	−11.75, 1.57				
**ABS-AWF**	Intercept	28.98 ***	22.71, 35.25	41.86	129.66	0.76	0.130/0.788
	Time	−6.8 **	−11.58, −2.02				
**ABS-co**	Intercept	38.44 ***	30.68, 46.20	102.64	160.08	0.61	0.163/0.673
	Time	−8.32 *	−15.76, −0.88				
**ABS-ach**	Intercept	36.41 ***	27.67, 45.15	113.28	220.04	0.66	0.172/0.718
	Time	−6.00	−13.84, 1.83				
**ABS-app**	Intercept	29.72 ***	20.69, 38.74	96.68	258.92	0.73	0.158/0.771
	Time	−6.97	−14.23, 0.29				
**ABS-IR**	Intercept	67.88 ***	53.89, 81.87	265.21	588.27	0.69	0.185/0.747
	Time	−10.11	−22.11, 1.89				
**ABS-R**	Intercept	36.7 ***	24.41, 48.99	234.12	424.66	0.64	0.137/0.693
	Time	−11.17	−22.43, 0.08				
**ABS II**	Intercept	104.58 ***	80.21, 128.96	825.36	1765.77	0.68	0.166/0.734
	Time	−21.41*	−42.58, −0.25				
**DAS-A**	Intercept	122.78 ***	102.48, 143.08	807.63	989.73	0.55	0.160/0.623
	Time	−7.52	−28.33, 13.29				
**ATQ**	Intercept	34.68 ***	29.19, 40.17	60.71	70.63	0.54	0.231/0.645
	Time	−7.89 **	−13.59, −2.18				
**CFS**	Intercept	15.9 ***	12.96, 18.84	26.82	10.79	0.29	0.322/0.517
	Time	−3.07	−6.82, 0.68				
**EQ5D3L**	Intercept	8.39 ***	7.74, 9.04	1.12	0.71	0.39	0.489/0.687
	Time	−0.86 *	−1.63, −0.09				
**EQ-VAS**	Intercept	61.4 ***	54.36, 68.45	170.12	46.27	0.21	0.339/0.481
	Time	13.57 **	4.17, 22.97				

Note: *** *p* < 0.001, ** *p* < 0.01,* *p* < 0.05; N_pre_ = 31, N_follow-up_ = 27; B—unstandardized regression estimate, 95% CI—95% confidence interval, σ^2^—residual variance, τ00—variance explained by random effects (subjects), ICC—intraclass correlation, r_m_^2^—marginal r squared (variance explained by fixed effects only), r_c_^2^—conditional r squared (variance explained by fixed and random effects), HARS = Hamilton anxiety rating scale, BDI = Beck Depression Inventory, ABS-DEM = ABS-II—demanding, ABS-SD/GE = ABS-II—self-downing/global evaluation, ABS-LFT = ABS-II—low frustration tolerance, ABS-AWF = ABS-II—awfulizing, ABS-co = ABS-II—comfort, ABS-ach = ABS-II—achievement, ABS-app = ABS-II—approval, ABS-IR = ABS-II—irrationality, ABS-R = ABS-II—rationality, ABS = attitudes and beliefs scale, DAS-A = dysfunctional attitudes scale, ATQ = automatic thoughts questionnaire, CFS = Chalder Fatigue Scale, EQ-5D-3L = health state and EQ-VAS = health rated on a visual analogue scale.

**Table 6:** Estimated marginal means.

	Pre-Test		Post-Test		Follow-Up	
	EM	SE	EM	SE	EM	SE
**HARS**	23.83	1.5	10.37	1.5	4.51	1.62
**BDI-II**	20.32	2.35	8.28	2.36	10.15	2.55
**ABS-DEM**	25.8	3.44	21.05	3.44	19.91	3.48
**ABS-SDG**	18.88	3.57	17.93	3.58	15.39	3.71
**ABS-LFT**	26.09	3.31	22.01	3.32	18.65	3.71
**ABS-AWF**	27.47	3.28	22.83	3.28	21.09	3.37
**ABS-co**	32.61	4.21	29.42	4.22	25.6	4.26
**ABS-re**	35.74	4.48	29.26	4.49	27.41	4.77
**ABS-ap**	29.85	4.64	25.54	4.65	21.92	4.88
**ABS-IR**	58.24	7.12	50.12	7.13	45.27	7.6
**ABS-R**	40.01	6.37	33.55	6.38	29.68	6.72
**ABS II**	98.25	12.75	84.18	12.76	74.84	13.26
**DAS-A**	131.57	10.1	107.35	10.11	110.43	11.22
**ATQ**	37.1	3.06	27.45	3.06	24.08	3.04
**CFS**	19.8	1.49	11.06	1.49	11.75	1.66
**EQ5D3L**	8.7	0.31	7.77	0.31	8.06	0.36
**EQ-VAS**	62.26	3.57	71.53	3.58	70.08	4.00

Note: EM—estimated mean, SE—standard error of mean, HARS = Hamilton anxiety rating scale, BDI-II = Beck Depression Inventory, ABS-DEM = ABS-II—demanding, ABS-SD/GE = ABS-II—self-downing/global evaluation, ABS-LFT = ABS-II—low frustration tolerance, ABS-AWF = ABS-II—awfulizing, ABS-co = ABS-II—comfort, ABS-ach = ABS-II—achievement, ABS-app = ABS-II—approval, ABS-IR = ABS-II—irrationality, ABS-R = ABS-II—rationality, ABS II = attitudes and beliefs scale, DAS-A = dysfunctional attitudes scale, ATQ = automatic thoughts questionnaire, CFS = Chalder Fatigue Scale, EQ-5D-3L = health state and EQ-VAS = health rated on a visual analogue scale.

**Table 7:** Significant effects of controlled variables.

Post-Test			
	Predictors	B	95% CI
ABSII-LFT	Sex	−12.23 *	−23.65, −0.81
	Time × Age	0.47 *	0.01, 0.94
ABSII-IR	Sex	−25.35 *	−49.89, −0.82
DAS-A	Time × MoCA	−10.82 *	−18.49, −3.14
CFS	Sex	6.45 *	1.32, 11.57
EQ5D3L	Time × Age	0.04 *	0.01, 0.08
Follow-up			
	Predictors	B	95% CI
ABSII-LFT	Sex	−12.24 *	−24.19, −0.29
ABSII-IR	Sex	−25.37 *	−50.21, −0.53
DAS-A	Time × MoCA	−9.2 *	−17.73, −0.67
ATQ	Time × MoCA	−2.51 *	−4.85, −0.17
CFS	Sex	6.45 *	1.23, 11.66
	Time × MoCA	−1.76 *	−3.29, −0.22
EQ5D3L	EDSS	0.63 ***	0.29, 0.96

Note: *** *p* < 0.001, * *p* < 0.05, B—unstandardized regression estimate and 95% CI—95% confidence interval.

## Data Availability

The data presented in this study are available on request from the corresponding author (cosmin.popa@umfst.ro).
